# Surgical Management of Huge Ranula Resembling Double Tongue in Pediatric Patients: A Successful Treatment of Two Cases

**DOI:** 10.7759/cureus.57884

**Published:** 2024-04-09

**Authors:** Fattirah Auni Fauzi, Mohd Faizal Abdullah, Norsamsu Arni Samsudin, Bazli Md Yusoff

**Affiliations:** 1 Oral and Maxillofacial Surgery, Hospital Universiti Sains Malaysia, Kota Bharu, MYS; 2 Oral and Maxillofacial Surgery, School of Dental Sciences, Universiti Sains Malaysia, Health Campus, Kota Bharu, MYS; 3 Pediatric Dentistry, Hospital Universiti Sains Malaysia, Kota Bharu, MYS; 4 Pediatric Dentistry, School of Dental Sciences, Universiti Sains Malaysia, Health Campus, Kota Bharu, MYS; 5 Radiology, Hospital Universiti Sains Malaysia, Kota Bharu, MYS

**Keywords:** case series, salivary glands, floor of mouth, surgical excision, ranula

## Abstract

Ranula is a fluid collection in a pseudo cystic wall secondary to the damage of the sublingual salivary gland causing blockage of salivary flow, leading to the extravasation phenomena. The growth rate of ranula varies depending on its severity. Due to its tendency to recur, the gold standard management of ranula has yet to be decided. The authors described two cases of young girls with huge ranulas on the floor of the mouth (FOM) resembling double tongue, which caused pain and discomfort during mastication. Following surgical excision of the ranulas along with the affected sublingual glands, both cases demonstrated successful treatment outcomes with no recurrence observed during post-operative follow-up. These cases highlight the importance of surgical excision of ranulas and removal of affected sublingual glands to prevent recurrence.

## Introduction

Ranula, a rare mucocele at the floor of the mouth (FOM) due to dehiscence of mylohyoid muscle in its anterior two-thirds, was observed in 45% of cadaveric study with major involvement from the sublingual gland, its duct of Rivinus and rarely from the surrounding minor glands [1,2]. Ranulas are classified into three types: simple ranulas have only intraoral swelling, plunging ranulas have submandibular swelling without swelling at the FOM, and mixed ranulas have both intraoral and neck swelling [3]. There is disagreement on the most effective treatment method for ranulas, which is a hotly debated topic. The surgical techniques range from aspiration to excision of the ranula along with the affected sublingual salivary gland. Some of the other techniques include marsupialization, dissection, cryotherapy, sclerotherapy, hydro-dissection, and LASER ablation. The rate of recurrence varies depending on the implemented procedure [4].

## Case presentation

Case 1

An 11-year-old girl came in with a three-month history of a painless, slow-growing swelling at her FOM. She had mild discomfort during mastication and a slight difficulty in communicating with her family members. However, the swelling was not associated with swallowing difficulties or pain. There was no stridor or tachypnea. She also denied any prior history of intraoral swelling, trauma, or neck swelling accompanied by difficulty in breathing, a change in voice, or noisy breathing. There was no family history of cancer.

**Figure 1:**
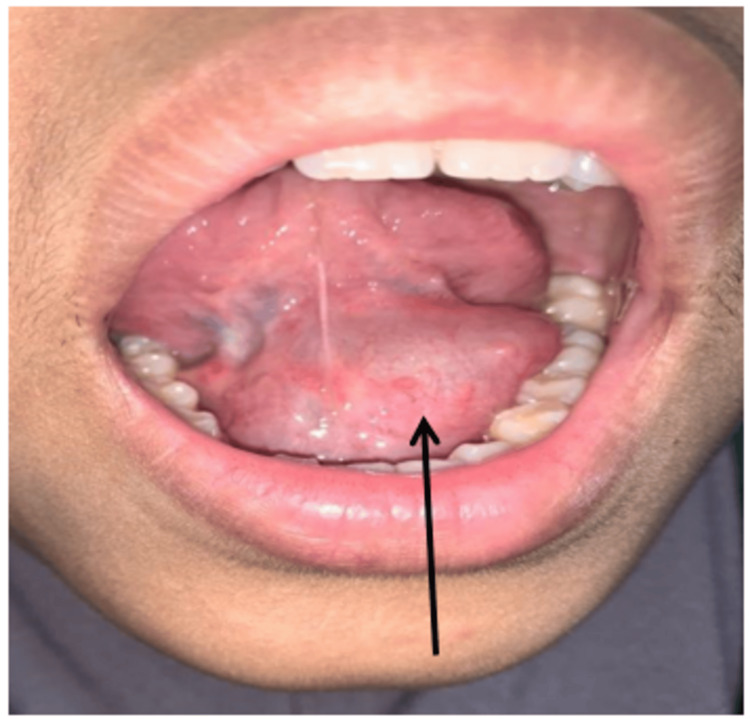
Swelling at FOM measuring 4x4 cm with double tongue appearance

A general examination revealed that the patient was alert and had no stridor or neck swelling. However, intraoral examination revealed huge bluish swelling measuring 4x4 cm on the FOM, causing upward displacement of the tongue with a resemblance of a double tongue appearance. The swelling was soft and non-tender, with no discharge noted (Figure 1).

**Figure 2:**
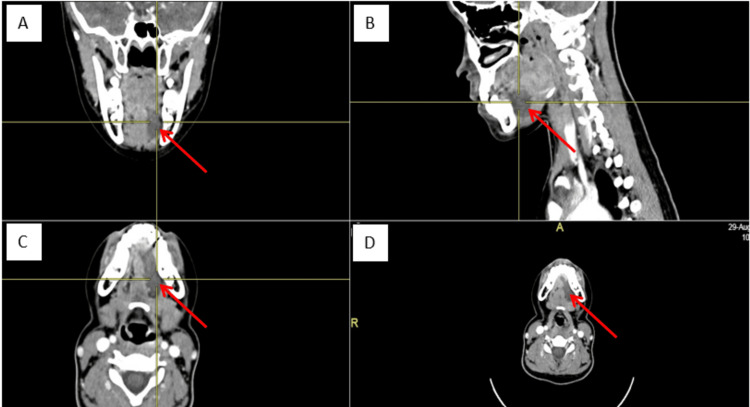
A-D: Coronal, sagittal, and axial view of CT scan revealed a hypodense lesion measuring 4x4 cm, confined to left sublingual space, which displaced left genioglossus medially, digastric muscle inferiorly, bounded laterally by mylohyoid muscle and abutted the base of the tongue superiorly A: Coronal view; B: Sagittal view; C-D: Axial view

Contrasted computed tomography (CT) scans revealed a well-defined, non-enhancing, homogeneous hypodense lesion at the left sublingual space with no enhancing solid component, fat component, or calcification within (Figure 2).

**Figure 3:**
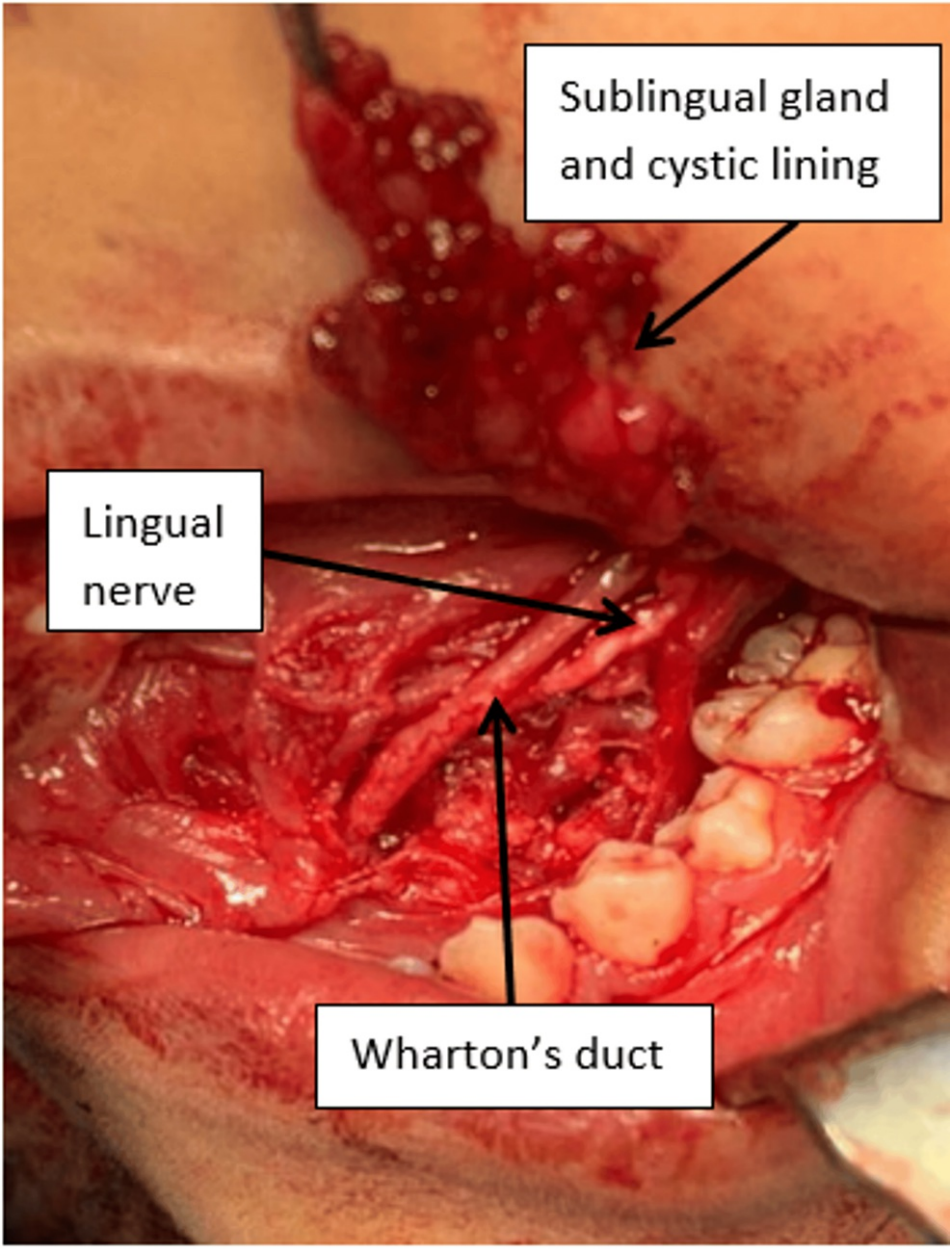
Removal of the left sublingual gland, the lingual nerve was seen crossing the Wharton duct from the lateral part to enter the tongue at the region of 36 and 35

A diagnosis of ranula was established. After a thorough explanation regarding treatment options for the ranula, both the patient and parents agreed to surgical excision of the ranula along with the affected left sublingual gland. Surgical excision was done under general anesthesia (GA). The patient was cleaned and draped after the injection of local anesthesia. Then, a lacrimal probe was utilized to locate the opening of the Wharton’s duct. After the successful cannulation of the duct, excision of the lesion was initiated on the lesion's superior surface, and the cystic lining, together with the left sublingual gland, was removed with both the Wharton’s duct and lingual nerve identified and preserved (Figure 3).

**Figure 4:**
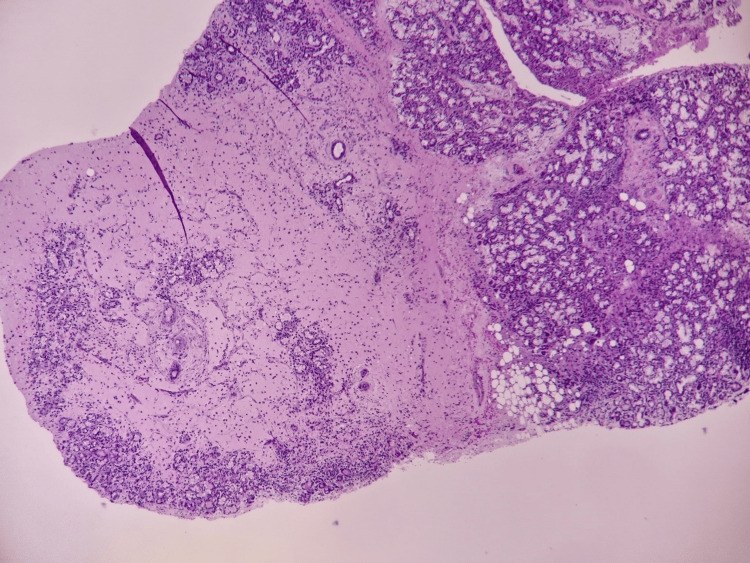
Extravasated mucin noted within the fibrous connective tissue stroma in between lobules of sublingual glands

The surgical specimen was sent for histopathological examination (HPE) which further confirmed the diagnosis of ranula (Figure 4).

Post-operative review of one week, one month, and six months revealed no recurrences, lingual nerve injury, Wharton’s duct injury, hematoma, wound dehiscence, and post-operative infection.

Case 2

An 11-year-old girl with underlying eczema was referred by a pediatric dentist for consultation on FOM swelling. The patient noticed the swelling started two weeks after she recovered from high-grade fever. The swelling gradually increased in size and was associated with pain. The pain score was 8/10 and was aggravated upon touching and during meals. The pain was throbbing in nature. However, the patient was able to tolerate orally and denied any trauma to the head and neck region, particularly at the lower lingual region. She claimed that this was the first episode of swelling. There was no stridor, snoring, or tachypnea episode at home. There was no history of malignancy in the family.

**Figure 5:**
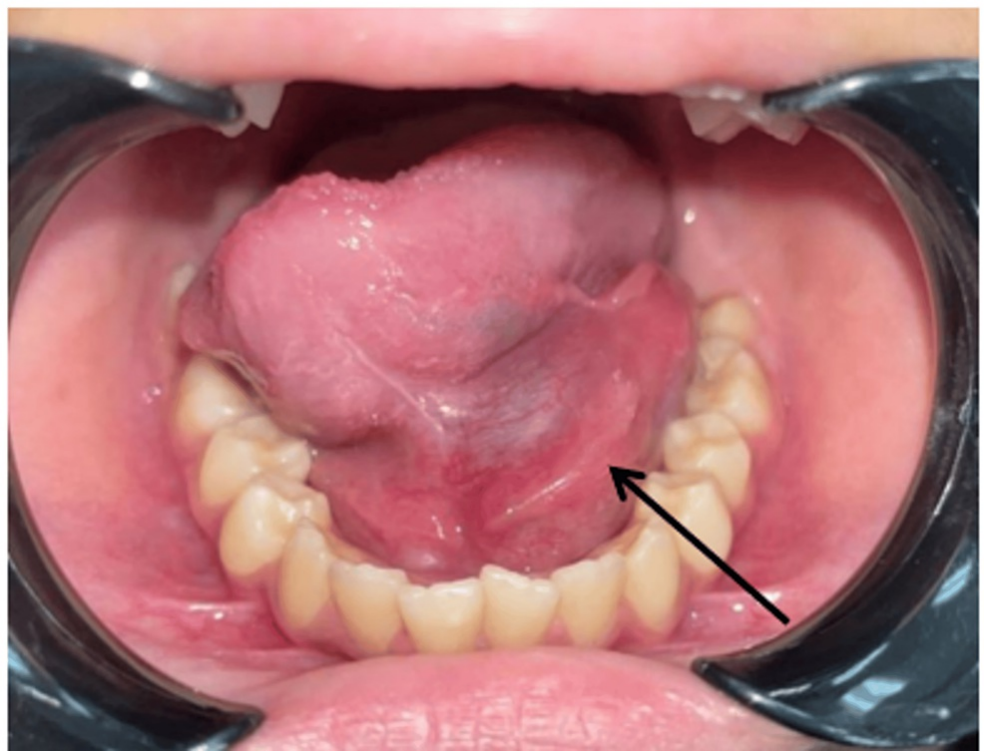
Swelling at FOM measuring 5x3 cm with double tongue appearance

Clinical examination revealed diffuse bluish dome-shaped swelling at the FOM measuring 5x3 cm in dimension (Figure 5).

**Figure 6:**
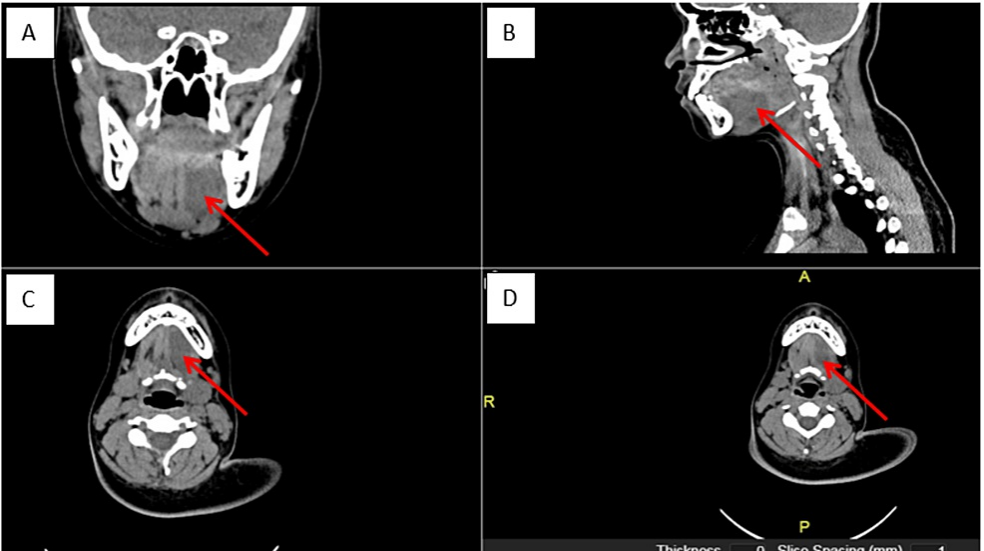
A-D: Cystic oval shape mass measuring 5x5 cm with no peripheral enhancement, located in the left sublingual space, bounded laterally by mylohyoid muscle, medially by genioglossus and posterolaterally by the submandibular gland A: Coronal view; B: Sagittal view; C-D: Axial view

A contrasted CT scan revealed a well-defined, non-enhancing, homogeneous hypodense lesion at the left sublingual space with the measurement of 5x4x2 cm with no enhancing solid component, fat component, or calcification within (Figure 6).

A diagnosis of ranula was established based on history, clinical examination, and radiographic findings.

**Figure 7:**
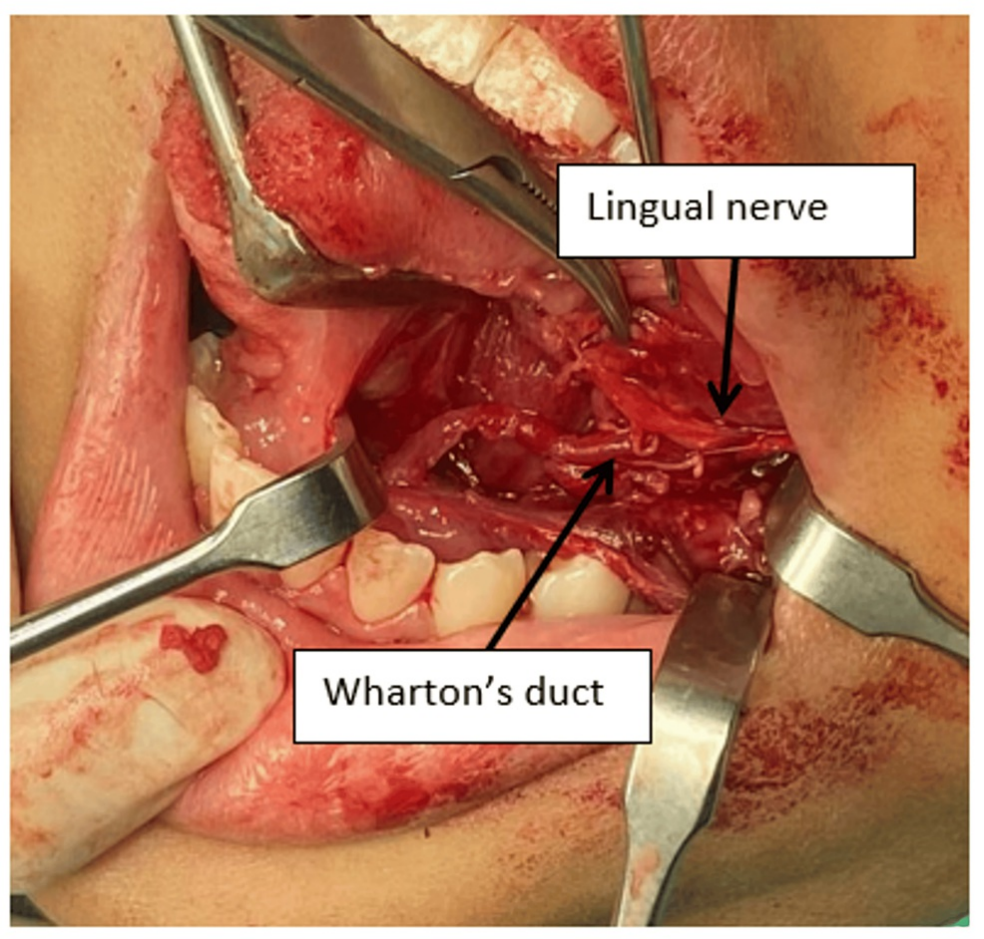
Lingual nerve and Wharton’s duct identified and preserved

After the patient was intubated, local anesthesia was administered at the left lingual region, and the left Wharton duct cannulation was successfully done using a lacrimal probe. An incision was made on the superior part of the lesion, and blunt dissection was done to separate the cystic lining from the normal underlying mucosa. Surgical excision of the ranula and left sublingual gland under GA was done with preservation of the left Wharton duct and lingual nerve (Figure 7).

**Figure 8:**
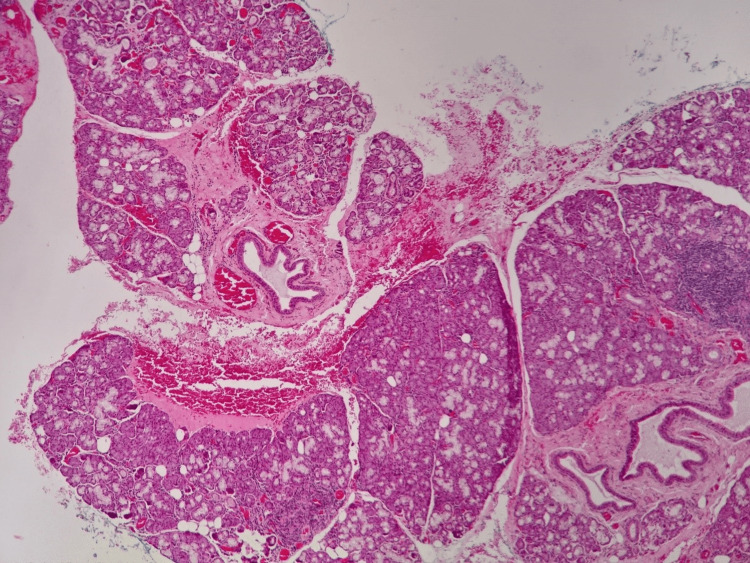
The sections show lobules of seromucous salivary acini with dilated ducts. Focal areas exhibiting lymphocytic infiltrate and acinar atrophy are seen. No extravasated mucin or sialoliths were observed within the given specimen

HPE result came out to be consistent with sialadenitis which coincides with the symptoms (Figure 8) and the diagnosis was revised as left sublingual gland sialadenitis.

## Discussion

Ranulas are pseudocysts that can be classified into retention or extravasation cysts and the latter is the most common type in which trauma acts as the initiating factor [5]. Left-side involvement of FOM with oral ranula was more frequent [3], as observed in both of our patients. The term ranula was derived from the Latin word rana, which means frog, and ranula, which describes a small frog due to its resemblance to its underbelly [6]. Studies have discovered a female gender preference for oral ranula, but there is no clear scientific basis for this [3,7]. Intra-oral ranulas are common in children and young adults [8]. Oral ranulas develop in the sublingual space above the mylohyoid muscle and are typically observed as a bluish cystic lesion at FOM.

Plunging or diving ranula occurs when the fluid pressure from the enlarging ranula travels through the mylohyoid muscle and into the submandibular or submental space [9]. Ranula typically manifests as a unilateral, painless mass in the FOM that grows slowly but can be bilateral, which is uncommon. Ranulas have an estimated prevalence of 0.2 cases per 1000 people and are responsible for 6% of salivary glands mucoceles [10]. In this case, both patients had massive diffuse swelling in the FOM with a lack of neck swelling, resembling a rare double tongue appearance. A large ranula can cause the tongue to be displaced superiorly and medially, resulting in airway obstruction, masticatory problems, dysphagia, and dyspnea [11]. Fortunately, in our cases, both patients had no signs of airway obstruction. Ludwig angina is frequently associated with diffuse swelling in the FOM involving both submandibular and submental spaces. With the history of high-grade fever in the second patient, a suspicion of odontogenic and salivary gland infection must be ruled out. However, the clinical cystic appearance of the lesion guides us to a diagnosis of ranula which later was revised to sialadenitis after being confirmed with HPE.

A surgical sieve is a method of differential diagnosis that prompts clinicians or surgeons to systematically consider various possibilities of swelling of the FOM. Differential diagnoses include infections, anatomic abnormalities during embryonic development, dermoid cysts, and vascular and lymphatic malformations. The infective cause is possible in our second case with pain and a history of high-grade fever. However, a clinical diagnosis was established with the help of radio imaging contrasted CT scan that confirmed the diagnosis of oral ranula. Dermoid cysts are another possibility, but they usually appear in the midline and grow slowly. The absence of lymphadenopathy in both cases and the benign nature of these lesions ruled out the sublingual salivary gland tumors. Vascular malformations around the head and neck region with a predilection to FOM are rare. Cavernous hemangioma occurs most commonly in the buccal space and FOM. Thus, a correct diagnosis is mandatory as this might affect the treatment's approach.

Imaging studies are often conducted to assess the extension of swelling before surgery or when the clinical diagnosis is uncertain for ranula [12].

Non-surgical and surgical treatments are available for ranula. Intracystic injection therapy with OK-432 and Botulin Toxin Type A is a non-surgical treatment option with a low recurrence rate [13]. Surgical treatments such as marsupialization, dissection, cryotherapy, sclerotherapy, hydro-dissection, and LASER ablation have been reported in the works of literature, with the recurrence rate varying according to the procedure performed [4]. Surgical excision of the ranula and sublingual glands was reported to yield zero recurrences [8]. However, several types of surgical intervention were proposed [1,2]: marsupialization with 13% recurrence, excision of ranula with 61% to 89% recurrence, incision and drainage with recurrence rate as high as 85%, and surgical excision with sublingual gland yield minimal recurrence rate of 3.6% [14]. Surgical interventions that omit the removal of the sublingual glands result in higher recurrence rates because they do not remove the source of pathology which is the sublingual gland [15]. On top of that, the recurrence rate depends more on the type of surgical intervention rather than the type of ranula [3]. Based on these findings, the parents decided to proceed with the surgical excision and removal of the sublingual gland for both cases. Post-operative reviews revealed no morbidity or recurrence. One potential limitation of the study is the small sample size, which may affect the generalizability of the findings. Additionally, the lack of long-term follow-up data limits the assessment of recurrence rates and the durability of the surgical interventions.

## Conclusions

Ranula is a common disease that causes swelling in the FOM. However, it can also cause chewing, swallowing, and airway obstruction if it grows exponentially large. It is usually diagnosed clinically, but other possibilities, such as infections and tumors, must be ruled out. In conjunction with radio-imaging investigations such as ultrasound, CT, or magnetic resonance imaging, history is useful in ruling out other possible diagnoses and providing an appropriate platform for management. Surgical intervention, which includes the removal of the ranula and the affected sublingual gland, may yield a zero recurrence rate and, therefore, can avoid unnecessary repeated surgical trauma, particularly in pediatric patients.
